# The Demographic Frame

**DOI:** 10.23889/ijpds.v9i5.2914

**Published:** 2024-09-18

**Authors:** Jennifer Ortman

**Affiliations:** 1 Census Bureau

The Demographic Frame is a comprehensive, secure, access-controlled database of person-level data consisting of demographic, social, and economic characteristics of individuals derived from census, survey, administrative, and third-party data sources. The Demographic Frame is facilitating innovations in data collection, processing, analyses, and products for census and survey programs.

